# Short- and Medium-Term Surgical Outcomes of Tissue-Engineered Pulmonary Valve Replacement in Sheep

**DOI:** 10.1007/s13770-025-00735-8

**Published:** 2025-07-07

**Authors:** Hussam Al Hussein, Hamida Al Hussein, Horatiu Suciu, David Emanuel Anitei, Carmen Sircuta, Ionela Cotfas, Bogdan Cordos, Cynthia Lefter, Klara Brinzaniuc, Dan Simionescu, Marius Mihai Harpa

**Affiliations:** 1 Department of Anatomy and Embryology, George Emil Palade University of Medicine, Pharmacy, Science and Technology of Targu Mures, 38 Gheorghe Marinescu Street, 540142 Targu Mures, Romania; 2 Doctoral School, George Emil Palade University of Medicine, Pharmacy, Science and Technology of Targu Mures, 38 Gheorghe Marinescu Street, 540142 Targu Mures, Romania; 3 Department of Surgery, George Emil Palade University of Medicine, Pharmacy, Science and Technology of Targu Mures, 38 Gheorghe Marinescu Street, 540142 Targu Mures, Romania; 4 Experimental Station, George Emil Palade University of Medicine, Pharmacy, Science and Technology of Targu Mures, 38 Gheorghe Marinescu Street, 540142 Targu Mures, Romania; 5 Regenerative Medicine Laboratory, CCAMF, George Emil Palade University of Medicine, Pharmacy, Science and Technology of Targu Mures, 38 Gheorghe Marinescu Street, 540142 Targu Mures, Romania; 6 Department of Cardiovascular Surgery, Emergency Institute for Cardiovascular Diseases and Transplantation, 50 Gheorghe Marinescu Street, 540136 Targu Mures, Romania; 7 Department of Anesthesiology and Critical Care, Clinical County Hospital Mures, 1 Gheorghe Marinescu Street, 540103 Targu Mures, Romania; 8 Department of Anesthesiology and Critical Care, Emergency Institute for Cardiovascular Diseases and Transplantation, 50 Gheorghe Marinescu Street, 540136 Targu Mures, Romania; 9 Department of Cardiology, Emergency Institute for Cardiovascular Diseases and Transplantation, 50 Gheorghe Marinescu Street, 540136 Targu Mures, Romania; 10 Biocompatibility and Tissue Regeneration Laboratory, Department of Bioengineering, Clemson University, Sikes Hall, Clemson, SC 29634 USA

**Keywords:** Tissue-engineered pulmonary valves, Cardiopulmonary bypass, Sheep model

## Abstract

**Background:**

Tissue-engineered pulmonary valves (TEPVs) hold considerable potential for improving outcomes in valve replacement surgeries. We investigated the surgical outcomes of TEPVs replacement in sheep, specifically examining the effects of valve type (decellularized versus adipose-derived stem cell-seeded valve [ADSC]) and the animal’s age at the surgery. The primary goals were to assess survival rates, postoperative complications, and the effects of cardiopulmonary bypass (CPB) on homeostasis.

**Methods:**

Nineteen juvenile and adult sheep were randomly assigned to orthotopic pulmonary valve replacement using either decellularized (DECELL, n = 10) or ADSC-seeded valves (CELL, n = 9). Blood gas analysis was conducted intraoperatively and postoperatively to assess CPB-related metabolic changes. The follow-up period after surgery was 6 months. Key demographic and operative parameters were recorded, and early and late postoperative complications were monitored.

**Results:**

No significant differences were observed in operative parameters or postoperative complications between the DECELL and CELL groups. Adult sheep exhibited longer anesthesia, CPB, and operative times due to tissue fragility but demonstrated better long-term survival than juveniles, who experienced more late-stage complications, including endocarditis. CPB exposure increased lactate and reduced hemoglobin levels, particularly in adult sheep, affecting homeostasis. The overall mortality rate was 42.1%, with deaths primarily attributed to congestive heart failure and endocarditis.

**Conclusion:**

Valve type did not significantly affect short-term outcomes and ADSC-seeding had no significant impact on operative parameters, postoperative complications, or survival rate. However, age remained a crucial factor influencing both surgical complexity and survival, highlighting the need for age-specific strategies in tissue-engineered valve applications.

## Introduction

Tissue bioengineering holds great promise for cardiovascular applications, aiming to create stem cell-populated scaffolds that replicate the native valves functions and offer regeneration, remodeling, and growth [1]. Sheep models, due to their physiological similarities to humans, are widely used to evaluate performance, surgical techniques, postsurgical complications, and overall efficacy of tissue-engineered heart valves (TEHVs) [2–4].

Decellularized scaffolds have played a critical role in TEHVs development, providing an intact extracellular matrix (ECM) that mimics the native valve properties while reducing the risk of immune rejection and enabling recellularization after implantation [5]. The first bioengineered pulmonary valve was implanted in 2000 in a 43-year-old patient, showing good hemodynamic performance and no major dysfunction [6]. The SynerGraft (CryoLife Inc.), a decellularized porcine valve, initially showed promise with successful implantation in sheep and humans, demonstrating cellular repopulation and intact ECM [7, 8]. However, severe inflammation, structural failure, and high mortality in pediatric patients due to incomplete decellularization and immune responses limited its clinical use [9]. Previous studies on TEHVs highlighted the potential of recellularization using various stem cell types, such as vascular-derived cells, bone marrow-derived cells, umbilical cord blood-derived cells [10, 11]. Cebotari et al. successfully implanted autologous-seeded human decellularized pulmonary valve allografts in pediatric patients, showing long-term growth adaptability and no valve degeneration over 3.5 years [12]. A few years later, transcatheter implantation of decellularized and reseeded biohybrid valves has emerged as a promising approach. However, Koenig et al. reported significant cell damage during the crimping process, emphasizing the need for further refinement of this technique [13].

Clinical trials, such as the ESPOIR trial, demonstrated the utility of decellularized allogenic scaffolds, reporting favorable outcomes, including good valvular performance and reduced explantation rates compared to traditional conduits [14]. Despite progress, challenges with xenogenic scaffolds remain, incomplete decellularization and immune responses leading to valve dysfunction and calcification. 

Cardiopulmonary bypass (CPB) has been shown to trigger inflammation, coagulation disturbances, and immune responses, providing insights into postsurgical complications. Prolonged CPB exposure worsens outcomes, increasing the risk of severe inflammatory reactions and potential organ dysfunction [15, 16]. In cardiac surgery involving CPB in large animal models like sheep, survival heavily depends on the quality of medical care during perioperative periods [17, 18]. Inadequate postoperative management and limited access to paraclinical investigations can increase mortality even more than the surgical technique itself [19]. Since John Gibbon’s first successful CPB procedure in 1953 [20], advancements in extracorporeal circulation (ECC) device technology and real-time monitoring during CPB have improved safety and patients outcomes by minimizing CPB’s impact [21].

This study presents the final surgical results of a project conducted at the Tissue Engineering and Regenerative Medicine Laboratory at the University of Medicine, Pharmacy, Science, and Technology “George Emil Palade” in Tîrgu Mures. In a previously published study, we outlined the anesthetic, surgical, and perioperative care protocols for orthotopic implantation of an acellular valve conduit in the pulmonary position on a beating heart in sheep [22].

In this study we evaluated the short- and medium-term surgical outcomes of orthotopic tissue-engineered pulmonary valves (TEPVs) implantation in sheep, using decellularized allografts and adipose-derived stem cell (ADSC) seeded valves. By focusing on postoperative complications, survival rates, and the effects of CPB, this study aimed to provide valuable data toward the clinical application of TEHVs. Furthermore, this work advances existing research by analyzing age-specific surgical outcomes and the physiological effects of CPB in a large-animal model. The study objectives were to evaluate: (1) the impact of valve type and animal age on surgical outcomes, (2) early and late postoperative complications, (3) CPB effects on sheep homeostasis, and (4) mortality rate and survival over a 6-month follow-up period.

## Materials and methods

### Animal selection

An experimental randomized study was conducted involving 19 juvenile and adult female sheep of the “Romanian Tsigaie” breed, purchased from a local farm. The sheep were randomly assigned to undergo orthotopic implantation of either decellularized pulmonary valves (DECELL, n = 10; adults n = 5, juveniles n = 5) or ADSC-seeded valves (CELL, n = 9; adults n = 4, juveniles n = 5), following the surgical protocol outlined in our previously published work [22]. Initially, the CELL group included 10 sheep, but one adult was excluded due to severe pneumonia following adipose tissue harvesting, reducing the group to nine. Upon acquisition, the sheep were housed in outdoor cages based on age and valve type. Before surgery, they were moved to an indoor facility with three individual recovery units designed for preoperative preparation and immediate postoperative care. The facility maintained optimal environmental conditions with controlled temperature (≈25 °C), humidity (≈60%), lighting, and ventilation. Postoperatively, sheep were housed in pairs to accommodate herd behavior and relocated to outdoor enclosures after 7–10 days. The sample size was determined based on prior experience, with the”VALVE REGEN” project, where similar experimental protocols achieved statistically significant and reproducible results. This approach aligns with the 3Rs principles (Replacement, Reduction, Refinement), ensuring ethical use of animals while maintaining scientific validity. Additionally, the 6-month follow-up period was chosen to balance the study's primary objectives with feasibility and ethical considerations, providing sufficient time to assess key outcomes without imposing unnecessary burdens on the animals.

Demographic data (age and body weight at acquisition and surgery), operative times (anesthesia, CPB, and total operative time), along with the type and frequency of early (within 7 days) and late (within 30 days) postoperative complications were recorded. The sheep were clinically evaluated and monitored before and after surgery throughout the follow-up period, as previously described [22]. In addition, laboratory investigations were performed. Blood gas analysis (ABL 90 FLEX Blood Gas Analyzer, Radiometer Medical, Denmark) was performed intraoperatively (pre-, during, and post-CPB) and postoperatively (day of surgery and day 7). The measured parameters included lactate, pH, hemoglobin (Hgb), hematocrit (Hct), serum potassium, and activated clotting time (ACT PLUS, Medtronic Inc. MN, USA).

### Preparation of the biological scaffold

The decellularized pulmonary valve scaffolds used in this study were prepared and seeded with ADSCs following the methods described by Movileanu et al. [23]. Pulmonary roots were obtained from freshly slaughtered sheep (30–40 kg) at a local slaughterhouse, extracted, and measured to match the designated animal’s body weight, minimizing the risk of valve mismatch. Decellularization was performed using a 10-day cyclic perfusion process with a pressure gradient of 20–25 mmHg, as described by Sierad et al. [24] and Movileanu et al. [25].

Autologous adipose tissue was harvested from the paravertebral region of nine sheep (CELL group) through minimally invasive procedures as detailed by Al Hussein et al. [26]. ADSCs were isolated as previously described by Zuk [27] and then differentiated into fibroblasts (FBs) and endothelial-like cells (ECs) and cryopreserved in dimethyl sulfoxide (DMSO) at − 140 °C (until seeding), following the methods detailed by Movileanu et al. [23, 28]. Each scaffold was seeded with 4 × 10^6^ FBs injected into the leaflets bases, and an additional 16 × 10^6^ FBs were dynamically seeded onto the adventitia using a rotational system for 48 h at one rotation per minute (rpm) in a cell culture incubator at 37 °C and 5% CO_2_. Similarly, 4 × 10^6^ ECs-like cells were statically incubated in the valve leaflets for 4 h, followed by intraluminal seeding of 16 × 10^6^ ECs and dynamic rotation at 1 rpm for 48 h. During the dynamic seeding phases the scaffolds were submerged in approximately 100 mL of sterile culture medium enriched with air from a CO_2_ incubator via an inline filter, in dedicated seeding chambers, ensuring cell viability and adhesion throughout the process [23]. 

Seeded valves underwent mechanical conditioning in a bioreactor, with gradually increasing pressure and perfusion frequency over five days, to simulate physiological pulmonary circulation. The conditioning protocol was as follows: Day 1: 10 mmHg at 17 beats per minute (bpm); Day 2: 15 mmHg at 30 bpm; Day 3: 19 mmHg at 45 bpm; Day 4–5: 24 mmHg at 60 bpm. This progressive conditioning ensured stable cell adhesion while preserving scaffold integrity, as validated in previous studies [23].

### Experimental procedures

The animal experimental procedures, including preoperative care, surgical technique, and postoperative management, were performed following the methods detailed in Al Hussein et al. [22]. A summary of the key steps is provided below:

*Preoperative Care:* On the day before surgery, the animals were weighed, shaved, cleaned, and fasted to ensure safety during anesthesia. They were clinically examined and laboratory parameters (blood gas, serum electrolytes, complete blood count, biochemical profile, coagulation tests) were evaluated also.

*Surgical Protocol:* Under general anesthesia a left lateral thoracotomy was performed through the third or fourth intercostal space. After systemic heparinization (250 IU/kg body weight), under normothermic CPB, the native pulmonary valve was excised. The decellularized pulmonary valve scaffold or ADSC-seeded valve was orthotopically implanted by termino-terminal anastomoses at the proximal level of the pulmonary trunk. 

*Postoperative Care:* Animals were observed continuously for the first 24 h after surgery to assess hemodynamic and respiratory stability. Subsequent monitoring included daily evaluations of consciousness, appetite, rumination, fecal and urine production, heart rate, SpO_2_, temperature, and wound healing. Blood gas analyses, serum electrolytes, and acid–base balances were performed regularly to identify and correct imbalances. Postoperative management included anticoagulation therapy with enoxaparin sodium, pain control with nonsteroidal anti-inflammatory drugs and analgesics, and prophylactic antibiotics to prevent infection.

### Study methodology

**Fig. 1:**
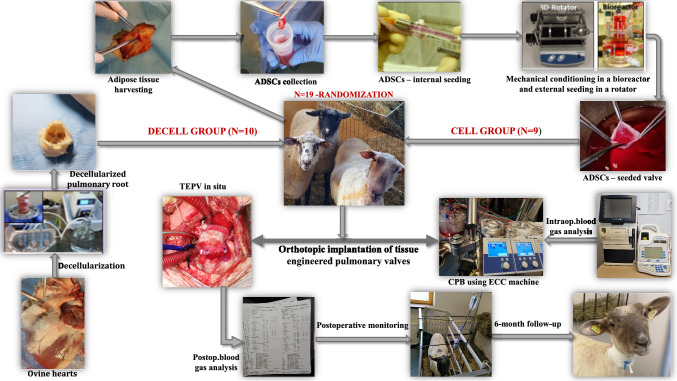
Study design. Legend: ADSCs, adipose-derived stem cells; TEPV, tissue-engineered pulmonary valve; CPB, cardiopulmonary bypass; ECC, extracorporeal circulation

The study design is presented in Fig. 1. Animals were randomly assigned into two groups to evaluate the impact of valve type on survival and postoperative complications:

- DECELL group (n = 10): Received a decellularized ovine pulmonary valve conduit.
- CELL group (n = 9): Received a decellularized pulmonary valve conduit seeded with ADSCs.

To assess the influence of age at surgery, animals were further categorized into:

- Juvenile group (n = 10): Younger than 24 months (DECELL, n = 5; CELL, n = 5).
- Adult group (n = 9): Older than 24 months (DECELL, n = 5; CELL, n = 4).

### Statistical analysis

Descriptive statistics (mean, median, and standard deviation) and inferential tests were used. Data distribution was assessed with the Shapiro–Wilk test. Group comparisons were conducted using the Student’s t-test with Welch correction for parametric data and the Mann–Whitney test for nonparametric data. Fisher’s exact or the chi-square tests were used to evaluate the associations between binary variables. Kaplan–Meier curves analyzed survival over the 6-month follow-up. The mortality rate was calculated as the percentage of deaths relative to the total number of animals used in the study. A *p* value < 0.05 was considered statistically significant, with a 95% confidence interval. Analyses were performed using GraphPad Prism software, version 10.3.1. (GraphPad Software, Boston, MA, USA).

### Ethical aspects

All procedures complied with the “Guide for the Care and Use of Laboratory Animals” and Directive 2010/63/EU on animal protection for scientific purposes. Euthanasia followed the Convention on Experiments on Live Vertebrate Animals and national regulations, including Law No. 9/2008 and Decision No. 19/2011 of the National Council of the College of Veterinarians. This study was approved by the Ethics Committee of the”George Emil Palade” University of Medicine, Pharmacy, Science, and Technology of Tîrgu Mures (approval no. 131/21.10.2016). The research adhered to the 3R principles (replacement, reduction, and refinement), minimizing the number of animals and ensuring proper analgesia during and after surgery to enhance animal welfare.

## Results

### Impact of valve substitute type on surgical outcomes

**Fig. 2:**
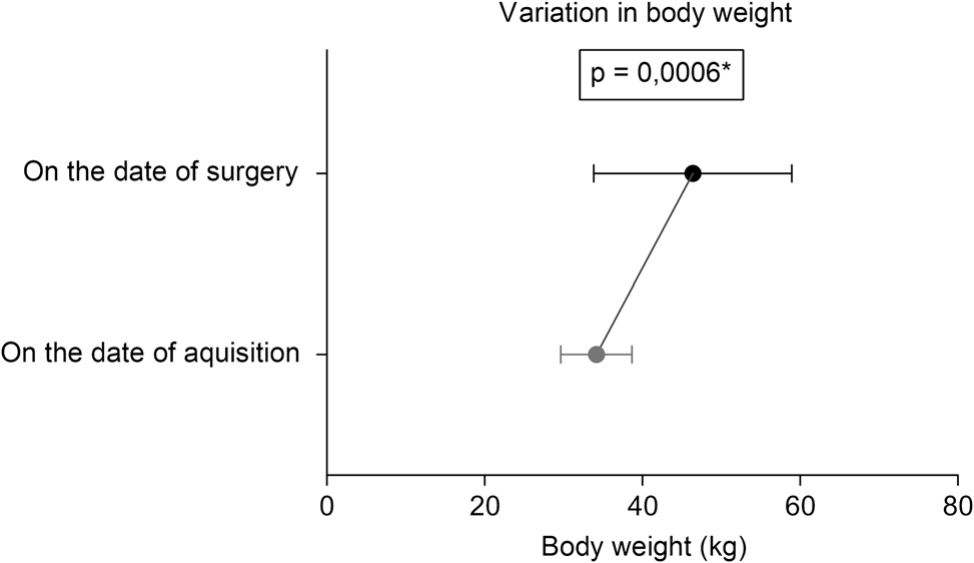
Differences in mean body weight between acquisition and surgery date

**Fig. 3:**
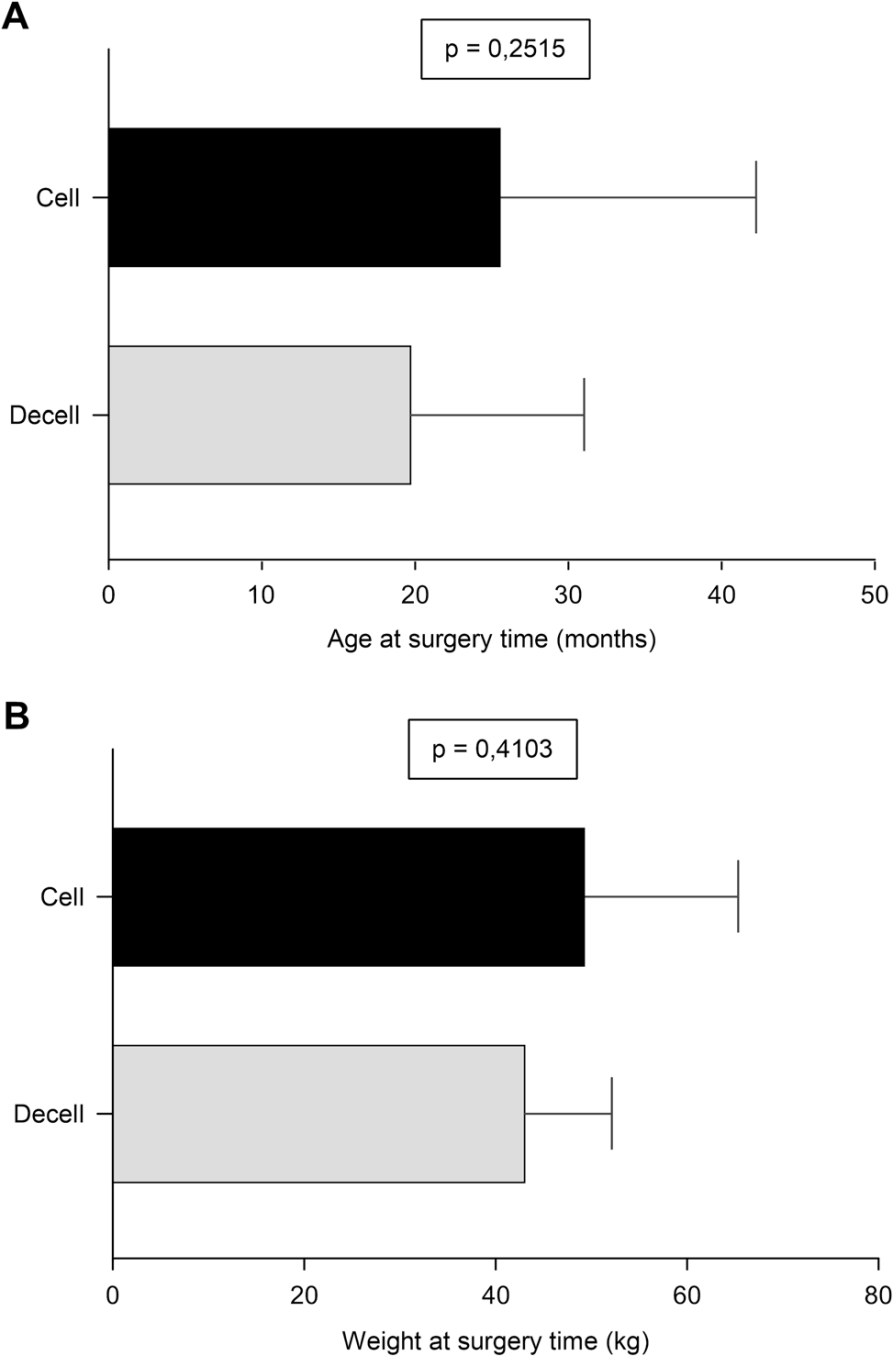
Comparative graphs show differences in **A** median age and **B** body weight between the DECELL and CELL groups

*Demographic Data and Operative Parameters*: The study included 19 female sheep, aged 7.8 to 44.3 months, with initial body weights ranging from 28.0 to 41.0 kg. During the 3–4 weeks preoperative preparation, the animals' weight increased by up to 57%, reaching 32.0–73.0 kg at surgery. A significant weight gain (*p* = 0.0006) was observed, reflecting the high level of preoperative care (Fig. 2). Demographic data and operative times for the DECELL and CELL groups are presented in Table 1. No significant differences were found in median age or body weight at surgery (Fig. 3A, B), although both tended to be higher in the CELL. This was expected, due to additional conditioning and seeding time required for ADSC-seeded valves. Anesthesia, CPB, and total operative times were comparable between the groups, suggesting that the intraoperative preparation of decellularized and ADSC-seeded valves does not prolong surgery.

**Table 1:** Demographic data and operative parameters according to valve substitute type

Variable	Total number of cases n = 19		*p* value
	DECELL (n = 10) Mean ± SD (Median)	CELL (n = 9) Mean ± SD(Median)	
Weight at acquisition (kg)	32.57 ± 4.69 (30.15)	35.97 ± 3.76 (34.50)	0.0788
Weight at the time of surgery (kg)	44.33 ± 8.75 (45.0)	49.67 ± 15.68 (39.00)	0.4103
Age at the time of surgery (months)	19.95 ± 11.10 (19.7)	25.79 ± 16.46 (13.1)	0.2515
Duration of anesthesia (min)	298.2 ± 32.34 (300.0)	293.6 ± 49.24 (270.0)	0.8090
Total operative time (min)	204.3 ± 26.98 (199.5)	203.6 ± 40.03 (197.0)	0.9623
CPB time (min)	65.80 ± 14.20 (64.50)	66.33 ± 14.04 (69.00)	0.9355
Thoracic drainage (mL)	380.0 ± 491.1 (250.0)	222.2 ± 271.7 (100.0)	0.1883

SD, standard deviation; CPB, cardiopulmonary bypass

*Postoperative Complications*: Were classified as early and late and further categorized into cardiac and noncardiac based on their manifestations (Tables 2 and 3). No significant differences were observed in the incidence of early or late complications between the DECELL and CELL groups.

**Table 2:** Early cardiac and noncardiac complications by valve substitute type

Manifestation	Total number of cases n = 19		*p* value
	DECELL (n = 10)	CELL (n = 9)	
*Early cardiac complications*			
Dyspnea (yes/no)	2/8	2/7	> 0.9999
Tachypnea (yes/no)	1/9	1/8	> 0.9999
Tachycardia (yes/no)	3/7	3/6	> 0.9999
Pleural effusion (yes/no)	0/10	1/8	0.4737
*Early noncardiac complications*			
Appetite (normal/impaired)	8/2	5/4	0,3498
Rumination (present/absent)	8/2	6/3	0,6285
Diuresis (present/absent)	9/1	9/0	> 0.9999
Fluid intake (normal/impaired)	10/0	7/2	0.2105
Bowel transit (present/absent)	9/1	5/4	0,1409
Tympanism (yes/no)	0/10	1/8	0.4737
Anemia (yes/no)	1/9	1/8	> 0,9999
Chills (yes/no)	1/9	0/9	> 0.9999
Hypothermia (yes/no)	1/9	0/9	> 0.9999
Hyperthermia (yes/no)	0/10	0/9	> 0.9999
Hypokalemia (yes/no)	2/8	0/9	0.4737
Hypocalcemia (yes/no)	2/8	0/9	0.4737
Hypomagnesemia (yes/no)	1/9	0/9	> 0.9999
Cough (yes/no)	0/10	3/6	0.0867

**Table 3:** Late cardiac and noncardiac complications by valve substitute type

Manifestation	Total number of cases n = 19		*p* value
	DECELL (n = 10)	CELL (n = 9)	
*Late cardiac complications*			
Dyspnea (yes/no)	3/7	4/5	0.6499
Tachypnea (yes/no)	0/10	0/9	> 0.9999
Tachycardia (yes/no)	2/8	1/8	> 0.9999
Bradycardia (yes/no)	0/10	1/8	0.4737
Pleural effusion (yes/no))	3/7	0/9	0.2105
Endocarditis (yes/no)	3/7	0/9	0.2105
*Late noncardiac complications*			
Appetite (normal/impaired)	9/1	7/2	0.5820
Fluid intake (normal/impaired)	10/0	8/1	0.4737
Bowel transit (present/absent)	9/1	9/0	> 0.9999
Hepatic dysfunction (yes/no)	2/8	0/9	0.4737
Tympanism (yes/no)	1/9	0/9	> 0.9999
Anemia (yes/no)	0/10	2/7	0.2105
Ascites (yes/no)	2/8	0/9	> 0.9999
Hyperthermia (yes/no)	3/7	1/8	0.5820
Pleural/pericardial effusion (yes/no)	1/9	0/9	> 0.9999
Cough (yes/no)	0/10	2/7	0.2105

### Age–specific surgical outcomes

**Fig. 4:**
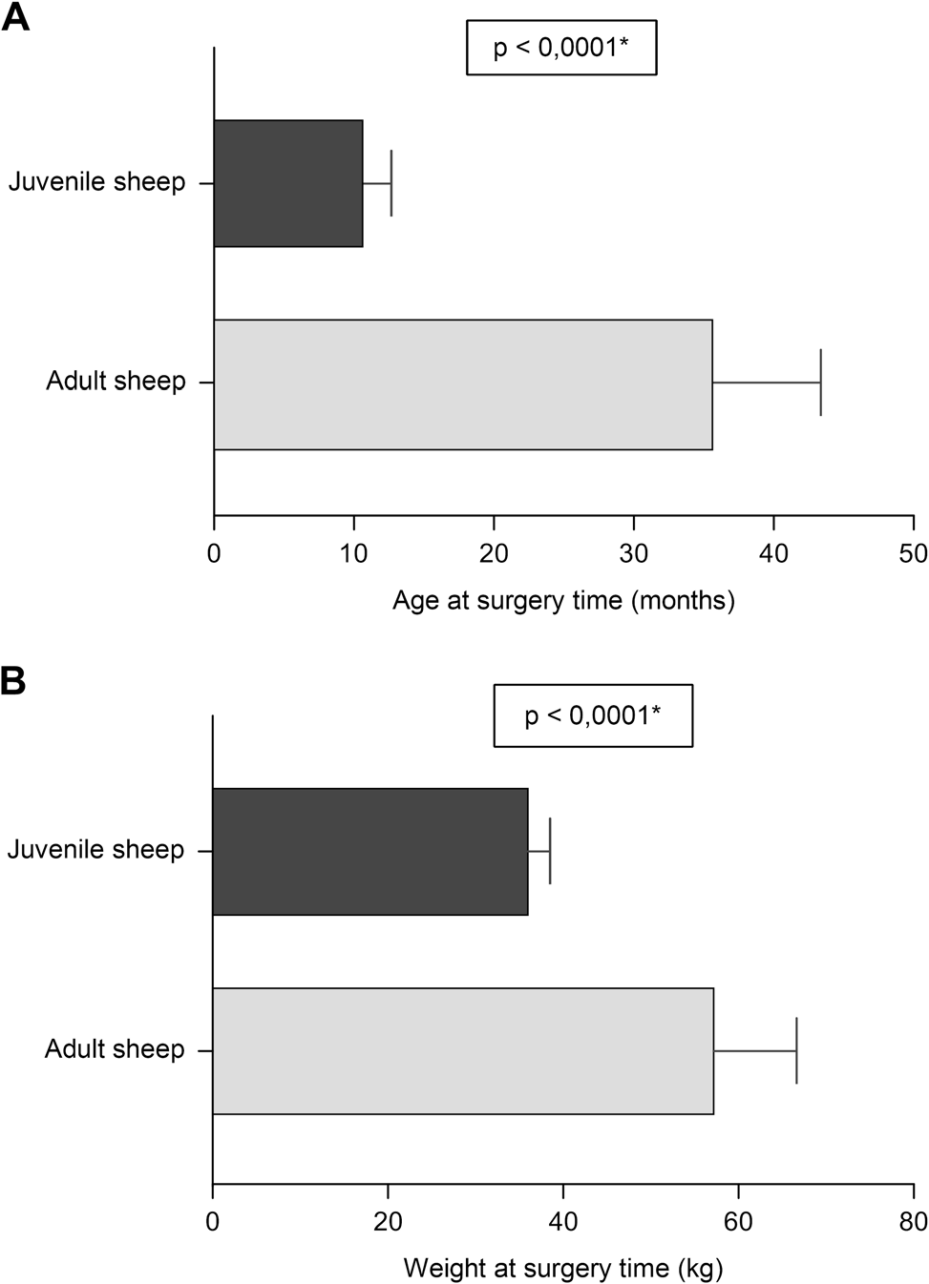
Comparative graphs show significant differences in **A** median age and **B** body weight between adult and juvenile sheep

**Fig. 5:**
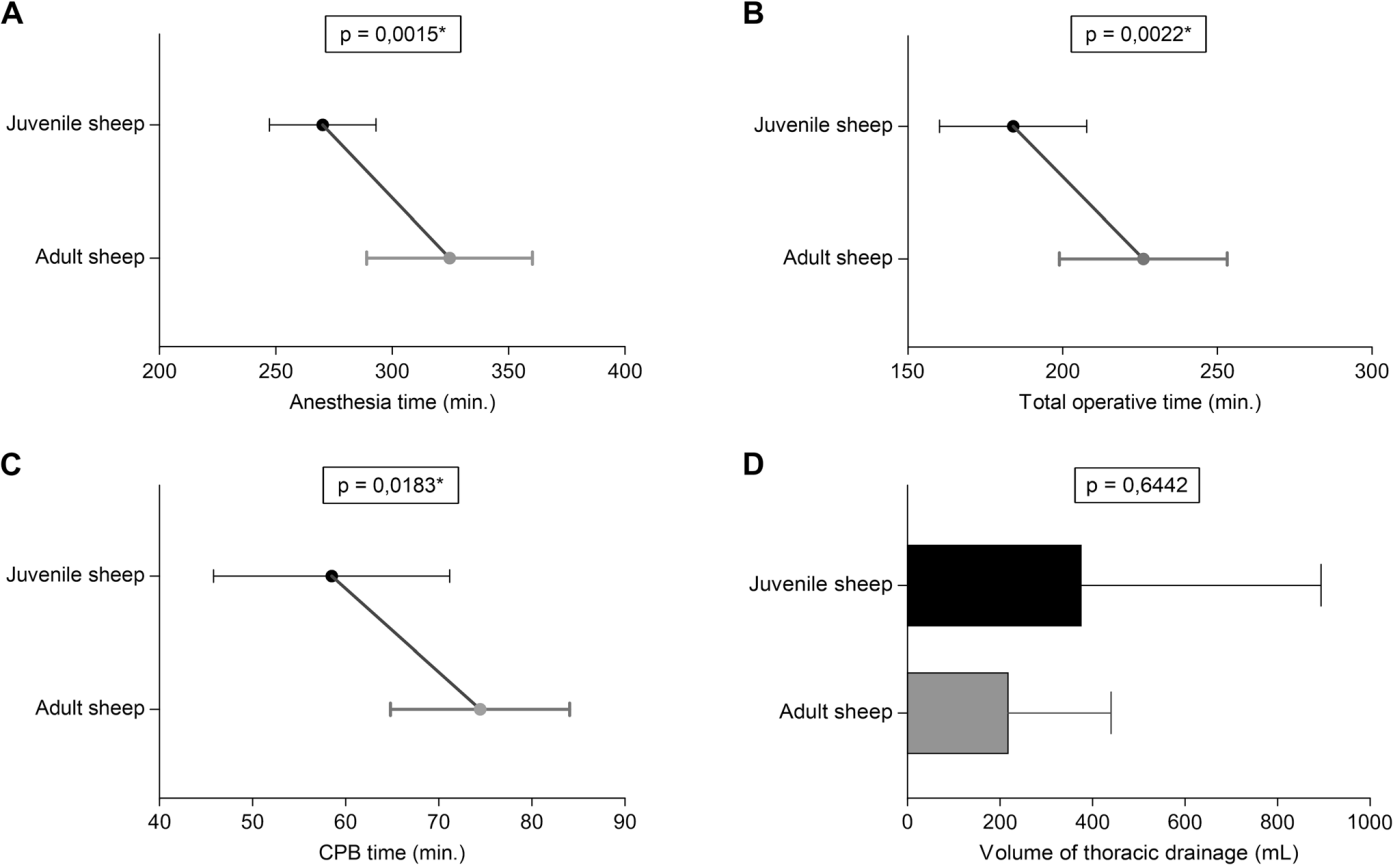
Comparative graphs show significant differences in **A** median anesthesia, **B** total operative and **C** CPB times, between adult and juvenile sheep; **D** no significant difference in postoperative fluid drainage volume. CPB, cardiopulmonary bypass

*Demographic Data and Operative Parameters*: Table 4 presents the demographic and operative data for adult (n = 9) and juvenile (n = 10) sheep. Significant differences were found in median age and weight at surgery (Fig. 4A, B), as well as in anesthesia, total operative, and CPB times (Fig. 5A–C). However, no significant differences were observed in median postoperative fluid drainage volumes (Fig. 5D).

**Table 4:** Demographic data and operative parameters by age group

Variable	Total number of cases n = 19		*p* value
	Adult sheep (n = 9) Mean ± SD (Median)	Juvenile sheep (n = 10) Mean ± SD (Median)	
Weight at acquisition (kg)	36.28 ± 5.64 (39.00)	32.29 ± 2.01 (32.65)	0.2358
Weight at surgery time (kg)	57.56 ± 9.08 (58.00)	36.36 ± 2.13 (36.50)	< 0,0001*
Age at surgery time (months)	35.87 ± 7.52 (37.5)	10.88 ± 1.81 (11.6)	< 0,0001*
Duration of anesthesia (min)	324.7 ± 3 5.62 (330.0)	270.2 ± 22.89 (272.5)	0,0015*
Total operative time (min)	226.1 ± 27.13 (220.0)	184.0 ± 23.77 (189.5)	0,0022*
CPB time (min)	74.44 ± 9.62 (75.00)	58.50 ± 12.69 (56.00)	0,0183*
Thoracic drainage (mL)	222.2 ± 218.1 (150.0)	380.0 ± 514.3 (250.0)	0,6442

*Postoperative Complications*: No significant differences were observed in the frequencies of early and late cardiac and noncardiac complications between adult and juvenile sheep, as presented in Tables 5 and 6.

**Table 5:** Early cardiac and noncardiac complications by age group

Manifestation	Total number of cases n = 19		*p* value
	Adult sheep (n = 9)	Juvenile sheep (n = 10)	
*Early cardiac complications*			
Dyspnea (yes/no)	2/7	1/9	0.5820
Tachypnea (yes/no)	2/7	0/10	0.2105
Tachycardia (yes/no)	2/7	4/6	0.6285
Pleural effusion (yes/no)	1/8	0/10	0.4737
*Early noncardiac complications*			
Appetite (normal/impaired)	7/2	6/4	0.6285
Rumination (present/absent)	8/1	6/4	0.3034
Diuresis (present/absent)	8/1	10/0	0.4737
Fluid intake (normal/impaired)	10/0	8/2	0.4737
Bowel transit (present/absent)	8/1	6/4	0.3034
Tympanism (yes/no)	1/8	0/10	0.4737
Anemia (yes/no)	0/9	2/8	0.4737
Chills (yes/no)	0/9	1/9	> 0.9999
Hypothermia (yes/no)	0/9	1/9	> 0.9999
Hyperthermia (yes/no)	0/9	0/10	> 0.9999
Hypokalemia (yes/no)	0/9	2/8	0.4737
Hypocalcemia (yes/no)	0/9	2/8	0.4737
Hypomagnesemia (yes/no)	0/9	1/9	> 0.9999
Cough (yes/no)	1/8	2/8	0.9999

**Table 6:** Late cardiac and noncardiac complications by age group

Manifestation	Total number of cases n = 19		*p* value
	Adult sheep (n = 9)	Juvenile sheep (n = 10)	
*Late cardiac complications*			
Dyspnea (yes/no)	2/7	5/5	0.3498
Tachypnea (yes/no)	0/9	0/10	> 0.9999
Tachycardia (yes/no)	1/8	2/8	> 0.9999
Bradycardia (yes/no)	0/9	1/9	> 0.9999
Pleural effusion (yes/no)	1/8	2/8	> 0. 9999
Endocarditis (yes/no)	0/9	3/7	0.2105
*Late noncardiac complications*			
Appetite (normal/impaired)	9/0	7/3	0.2105
Fluid intake (normal/impaired)	9/0	9/1	> 0.9999
Bowel transit (present/absent)	9/0	9/1	> 0.9999
Hepatic dysfunction (yes/no)	0/9	2/8	0.4737
Tympanism (yes/no)	0/9	1/9	> 0.9999
Anemia (yes/no)	1/8	1/9	> 0.9999
Ascites (yes/no)	0/9	2/8	0.4737
Hyperthermia (yes/no)	2/7	2/8	> 0.9999
Pleural/pericardial effusion (yes/no)	0/9	1/9	> 0.9999
Cough (yes/no)	1/8	1/9	> 0.9999

### Effects of cardiopulmonary bypass on ovine homeostasis

The parameters measured through blood gas analysis to evaluate the impact of CPB on acid–base balance, hydroelectrolytic equilibrium, and secondary anemia are presented in Table 7. Additionally, the ACT values recorded at various stages of the procedure are shown in Table 8.

**Table 7:** Parameters measured through blood gas analysis at different stages of the procedure

Stage	Total number of cases n = 19		*p* value
	Adult sheep (n = 9) Mean ± SD (Median)	Juvenile sheep (n = 10) Mean ± SD (Median)	
*Lactate (mmol/L)*			
Intraoperative pre-CPB	9.77 ± 5.19 (7.00)	4.70 ± 2.50 (4.00)	0.0214*
Intraoperative during CPB	12.25 ± 4.46 (11.50)	3.71 ± 1.11 (4.00)	0.0012*
Intraoperative post-CPB	23.44 ± 7.14 (23.00)	14.40 ± 8.42 (12.50)	0.0215*
Surgery day	25.22 ± 21.60 (13.00)	29.80 ± 27.89 (25.50)	0.7434
7th postoperative day	10.75 ± 7.65 (8.50)	15.00 ± 8.19 (13.00)	0.1801
*Blood pH*			
Intraoperative pre-CPB	7.43 ± 0.05 (7.43)	7.44 ± 0.05 (7.44)	0.4262
Intraoperative during CPB	7.49 ± 0.11 (7.46)	7.44 ± 0.06 (7.45)	0.2368
Intraoperative post-CPB	7.47 ± 0.12 (7.45)	7.44 ± 0.08 (7.41)	0.5379
Surgery day	7.36 ± 0.07 (7.34)	7.37 ± 0.09 (7.34)	0.6630
7th postoperative day	7.51 ± 0.04 (7.50)	7.51 ± 0.05 (7.51)	0.8940
*Hemoglobin (g/dL)*			
Intraoperative pre-CPB	8.66 ± 0.65 (8.60)	9.24 ± 1.08 (9.20)	0.1767
Intraoperative during CPB	7.70 ± 1.54 (7.90)	9.41 ± 0.96 (9.20)	0.0229*
Intraoperative post-CPB	7.87 ± 1.60 (7.90)	8.35 ± 1.24 (8.15)	0.4697
Surgery day	7.71 ± 1.11 (7.70)	9.30 ± 0.90 (9.30)	0.0032*
7th postoperative day	10.39 ± 1.21 (10.55)	10.63 ± 1.32 (11.00)	0.6929
*Hematocrit (%)*			
Intraoperative pre-CPB	26.56 ± 1.97 (26.50)	28.56 ± 3.13 (28.25)	0.1184
Intraoperative during CPB	23.60 ± 4.45 (24.15)	28.89 ± 2.92 (28.30)	0.0223*
Intraoperative post-CPB	23.80 ± 4.96 (23.60)	25.04 ± 4.20 (24.20)	0.5627
Surgery day	29.69 ± 4.06 (29.50)	28.84 ± 3.50 (28.60)	0.6308
7th postoperative day	30.34 ± 4.48 (29.40)	31.02 ± 5.14 (32.50)	0.7712
*Serum potassium levels (mmol/L)*			
Intraoperative pre-CPB	3.21 ± 0.52 (3.10)	3.15 ± 0.46 (3.25)	0.7896
Intraoperative during CPB	3.05 ± 0.41 (3.00)	3.00 ± 0.44 (3.10)	0.8234
Intraoperative post-CPB	3.70 ± 0.72 (3.40)	3.82 ± 0.69 (4.00)	0.7170
Surgery day	3.45 ± 0.54 (3.50)	3.21 ± 0.68 (3.30)	0.4034
7th postoperative day	4.11 ± 0.23 (4.10)	4.02 ± 0.42 (3.95)	0.5693

**Table 8:** Coagulation homeostasis parameters by age group

Variable	Total number of cases n = 19		*p* value
	Adult sheep (n = 9) Mean ± SD (Median)	Juvenile sheep (n = 10) Mean ± SD (Median)	
Heparin dose IV (mg/kg)	2.98 ± 0.61 (2.88)	4.18 ± 0.94 (4.23)	0.0043*
Heparin dose in the ECC circuit (mg/kg)	1.12 ± 0.20 (1.00)	1.34 ± 0.49 (1.23)	0.1972
Total dose of heparin (mg/kg)	4.10 ± 0.53 (3.85)	5.53 ± 1.28 (5.36)	0.0068*
ACT pre-CPB (s)	99.78 ± 21.07 (99.0)	122.60 ± 15.17 (124.0)	0.0175*
Protamine dose (mg/kg)	3.59 ± 0.88 (3.44)	5.04 ± 3.23 (4.06)	0.2015
ACT post-CPB (s)	113.0 ± 17.76 (107.0)	226.8 ± 355.9 (121.5)	0.1074

ECC, extracorporeal circulation; CPB, cardiopulmonary bypass; ACT, activated clotting time; IV, intravenous

**Fig. 6:**
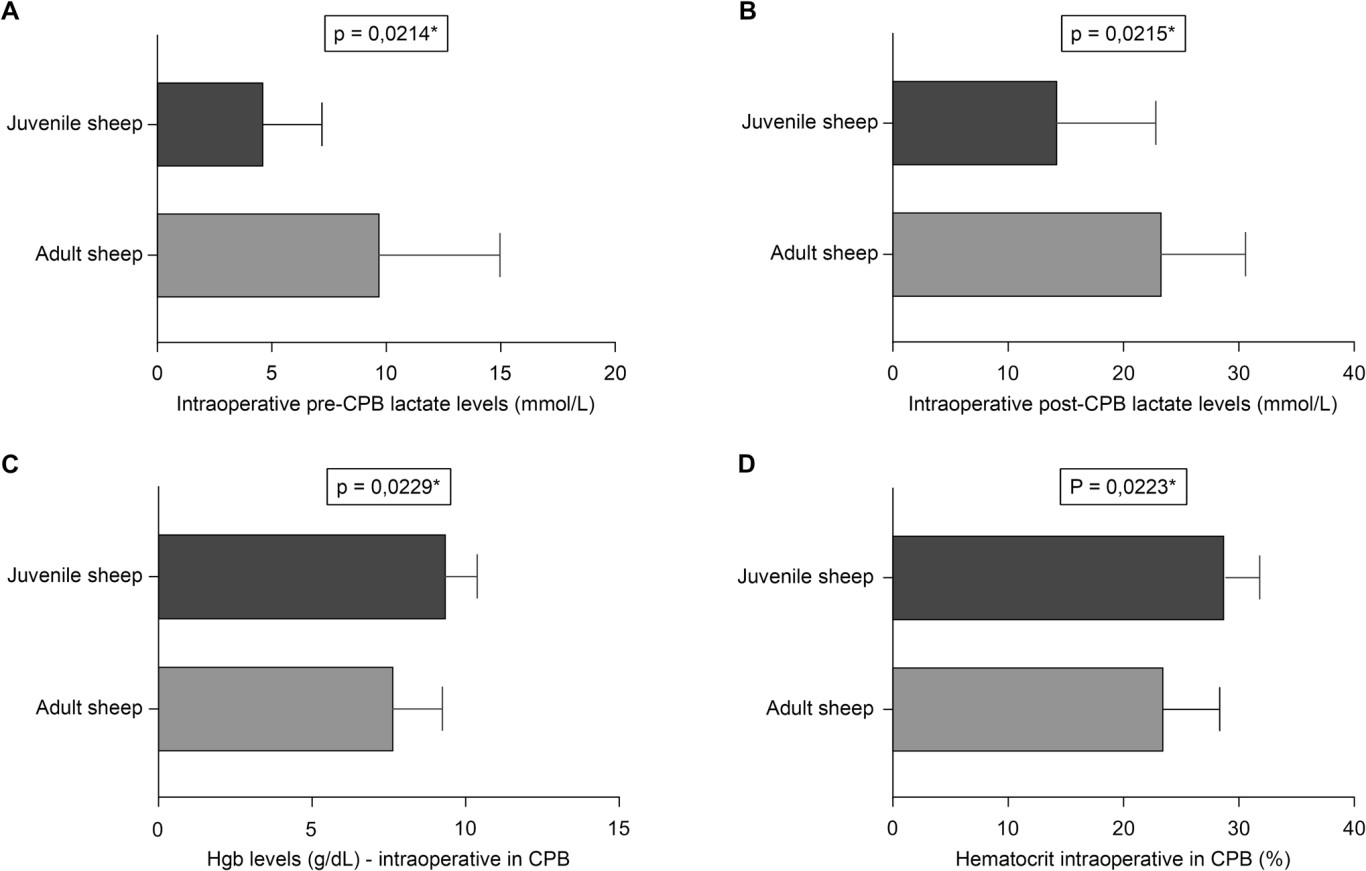
Comparative statistical analysis of intraoperative parameters between adult and juvenile sheep: **A** pre-CPB lactate levels; **B** post-CPB lactate levels; **C** hemoglobin levels during CPB; **D** hematocrit levels during CPB. CPB, cardiopulmonary bypass

*a) Lactate*: Significant differences in lactate levels pre-, during, and immediately post-CPB were identified between adult and juvenile sheep (Fig. 6A, B).

*b) Hemoglobin and Hematocrit*: Significant differences between adults and juveniles were observed for hemoglobin and hematocrit during CPB exposure (Fig. 6C, D), and in hemoglobin levels on the day of surgery (Table 7).

*c) Blood pH*: No significant differences in blood pH were observed between the two groups at any intraoperative or postoperative stage.

**Fig. 7:**
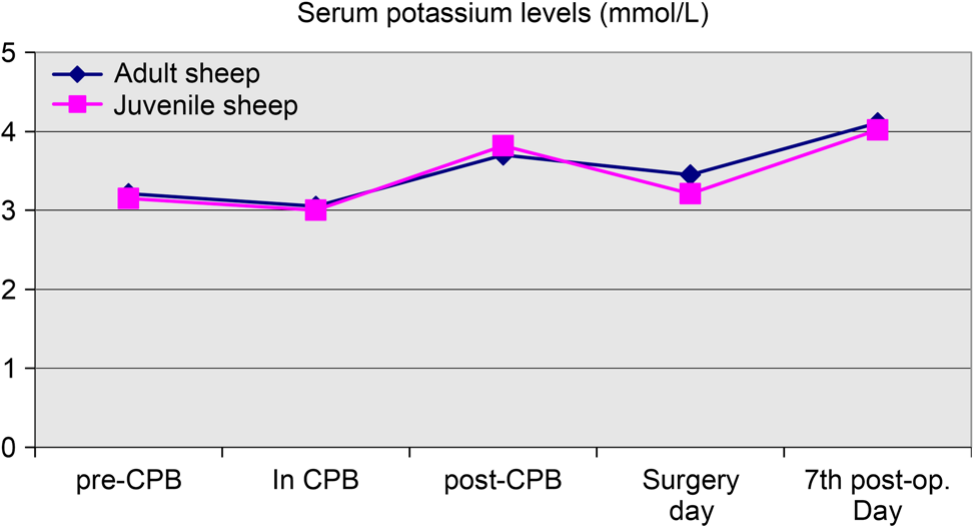
Comparative graphs of intraoperative and early postoperative potassium levels between adult and juvenile sheep

*d) Serum Potassium Levels*: Statistical analysis showed no significant differences between the mean potassium levels of the two groups (*p* > 0.05) (Fig. 7).

**Fig. 8:**
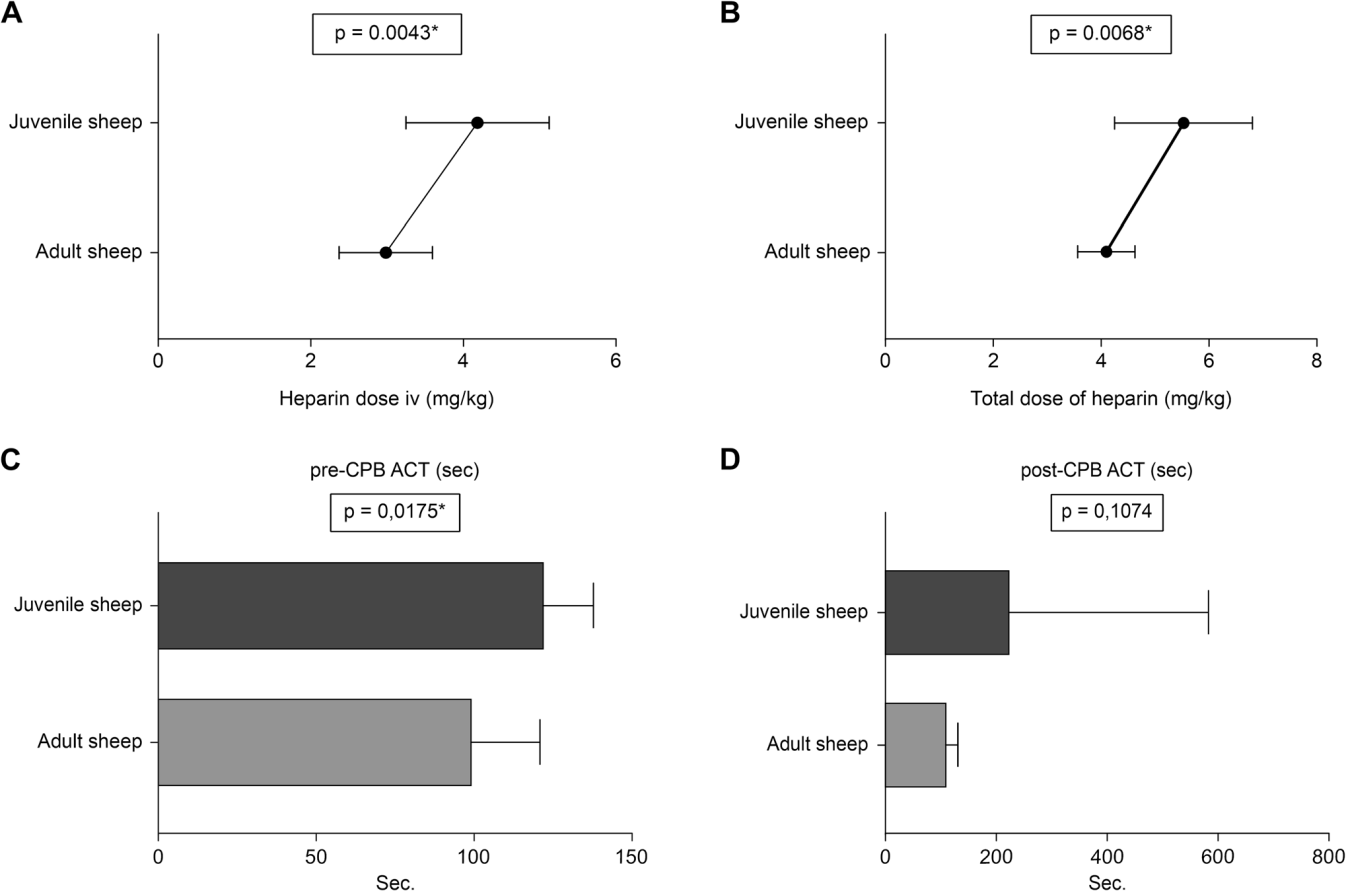
Comparative graphs of coagulation parameters between adult and juvenile sheep: **A** intravenous heparin dose (mg/kg); **B** total dose of heparin (mg/kg); **C** pre-CPB ACT (s); **D** post-CPB ACT (s). CPB, cardiopulmonary bypass

*e) Coagulation Homeostasis*: Table 8 presents data on heparin and protamine doses, as well as pre- and post-CPB ACT values. Significant differences were observed between adult and juvenile sheep in intravenous heparin dose (Fig. 8A), total heparin administered (Fig. 8B), and pre-CPB ACT values (Fig. 8C), while post-CPB ACT values showed no significant differences (Fig. 8D). The heparin-to-protamine ratio for heparin reversal was 1:0.89 in adult and 1:0.76 in juvenile sheep.

### Fatality rate and survival

**Fig. 9:**
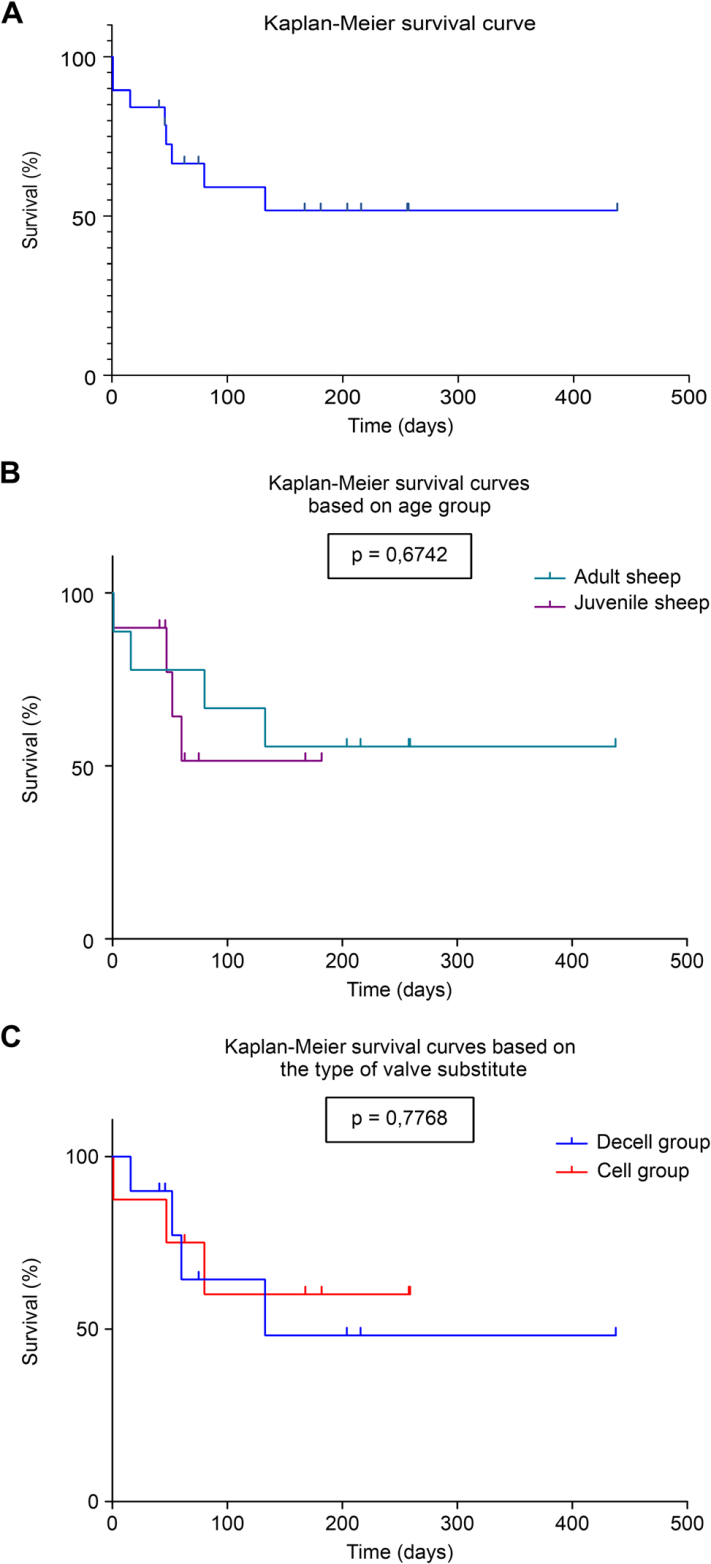
**A** Kaplan–Meier survival curve for the entire experimental animal cohort (n = 19); **B** comparative survival curves between adult and juvenile sheep; **C** comparative survival curves between the DECELL and CELL group

The incidence of deaths and their causes are presented in Table 9. The overall fatality rate, was 42.1%. The Kaplan–Meier survival curve (Fig. 9A) shows survival rates of 84.2% at 1 month, 68.4% at 2 months, 63.2% at 3 months, and 57.9% at 6 months. One animal was monitored for > 6 months (62 weeks) to assess the feasibility of extended follow-up in future large animal studies.

**Table 9:** Surgical complications, incidence of deaths, and their causes

Animal number	Preoperative complications	Intraoperative complications	Complications within the first 7 days	Late complications	Death/Survival	Cause of death/euthanasia
*Decell Group*						
1		–	–	–	Survived	–
2	- Arrhythmia, HR 75 bpm	- Episodes of PH, high BP upon exit from CPB	–	–	Survived	–
3	–	–	–	–	Survived	–
4	–	–	- Appetite, rumination, and diuresis affected - Dyspnea	- Pericardial effusion and left pleural effusion - Fever	Death on day 16	- Cardiac tamponade due to conduit rupture - Possible endocarditis
5	–	–	- Dyspnea - Tachypnea	–	Death in week 19	-CHF - Severe dyspnea
6	- Respiratory dysfunction - Cough and inspiratory stridor	–	- Appetite, rumination affected -Hypothermia	- Small pericardial and pleural effusion - Endocarditis (on TTE) - Ascites (on abdominal ultrasound) - Fever - E. coli and enterococcus detected in blood culture	Unscheduled euthanasia in week 6	-CHF
7	- Spontaneous epistaxis from the right nostril	–	- CPA resuscitated through ECM after sedation for TTE	- Liver dysfunction - tachycardia - Fever - general condition deteriorated over the last 3 weeks	Death in week 8	- Sudden death during transfer to the new shelter - Endocarditis with thrombi and vegetations on the explanted valve
8	–	–	- Intermittent tachycardia	- Ascites in a large amount - Liver dysfunction - Dyspnea	Unscheduled euthanasia in week 11	-CHF - Generalized edema - Large pleural effusion
9	–	–	- Intermittent tachycardia - Hypokalemia - Hypocalcemia - Hypomagnesemia - Impaired intestinal transit	- tympanism (in the last month) - Impaired intestinal transit - Decreased appetite - E. coli and enterococcus detected in blood culture (endocarditis) - Intermittent tachycardia - Progressively worsening dyspnea in the last month	Unscheduled euthanasia in week 7	-CHF - Severe dyspnea - Large pleural effusion
10	–	–	- Intermittent tachycardia - Anemia - Hypokalemia - Hypocalcemia	- Pronounced dyspnea in the last month - Small pericardial effusion (on TTE) - Endocarditis suspicion on TTE - Large pleural effusion, drained via pleural puncture - Altercation with shelter mates resulting in a proximal tibia fracture	Death in week 9	- CHF - Severe dyspnea - Large pleural effusion
*Cell group*						
1	–	- CPA during wound chest closure	–	–	Intraoperative death	- Aortic wall rupture - Hypovolemic shock
2	–	- Aortic wall rupture at decannulation, resolved through aortic suturing	- Mild dyspnea - Intermittent tachypnea - Small basal pleural effusion - Impaired/decreased appetite - Irritating cough	- Mild dyspnea - Intermittent tachycardia - Subfebrile condition - Irritating cough	Survived	–
3	–	- Aortic wall rupture at decannulation, resolved through aortic suturing	- Intermittent tachycardia - Impaired intestinal transit - tympanism	–	Survived	–
4	–	–	- Marked tachycardia	- Marked dyspnea in the last week - Respiratory failure - Submandibular abscess	Death in week 12	- Severe heart failure - Respiratory failure - Anasarca
5	–	–	–	–	Death at 4 h postoperatively	- Pneumothorax after the animal pulled out the drainage tube (psychotic agitation)
6	tracheobronchial secretion - PH	- Severe uncontrollable hypercapnia at the end of the operation	- Persistent tachycardia - Appetite, rumination, water intake, and intestinal transit affected - Irritating/productive cough - Repeated vomiting	- Pronounced dyspnea in the last week - Respiratory failure - Decreased appetite and water intake in the last week - Anemia - Intermittent cough - Anaphylactic shock after administration of iv Iron (III)-hydroxide sucrose complex 100 mg/5 ml	Unscheduled euthanasia in week 9	- Severe heart failure - Respiratory failure - Anasarca
7	–	–	- Appetite, rumination, water intake, and intestinal transit affected on the 1st day - Irritating cough on the 1st day	- Intermittent bradycardia - Mastitis drained on day 17	Survived	–
8	–	–	- Anemia	-	Survived	–
9	–	–	- Absent rumination on the 1st and 2nd day postoperatively - Decreased intestinal transit on the 1st and 2nd day postoperatively	- Decreased appetite in the last week - Progressively worsening dyspnea - Respiratory failure - Altered general condition in the last week	Death in week 7	- Heart failure and severe dyspnea - Respiratory failure

HR, heart rate; PH, pulmonary hypertension; BP, blood pressure; CPB, cardiopulmonary bypass; CPA, cardiopulmonary arrest; ECM, external cardiac massage; TTE, transthoracic echocardiography; CHF, congestive heart failure; IV, intravenous

Figure 9B compares the survival curves of adult and juvenile sheep, with rates of 77.8% vs. 90% at 1 month, 77.8% vs. 64.3% at 2 months, 66.7% vs. 51.4% at 3 months, and 55.6% vs. 51.4% at 4 months, remaining stable until the end of the follow-up. No significant differences were found between the groups (*p* > 0.05, Mantel–Cox chi-square test).

Figure 9C compares survival rates between DECELL and CELL groups, with rates of 90% vs. 87.5% at 1 month, 77.1% vs. 75% at 2 months, 64.3% vs. 60% at 3 months, and 48.2% vs. 60% at 4 months, remaining stable thereafter. No significant differences were observed between the two groups (*p* > 0.05). 

The causes of death or euthanasia before the scheduled date, along with preoperative, intraoperative, and both early and late postoperative complications, are detailed in Table 9.

## Discussion

Since 2000, efforts have focused on developing living heart valves through tissue engineering.

Decellularized scaffolds have shown reduced immunogenicity and favorable outcomes in animal models, but the benefits of recellularization remain debated. Limited comparative studies report inconsistent results. Tudorache et al. demonstrated that seeding decellularized aortic valves with mature ECs isolated from jugular veins prevented calcification, inflammation, and degeneration, yielding excellent hemodynamic outcomes in young sheep [29]. Conversely, Theodoridis et al. reported no functional or structural improvements using ECs-like cells differentiated from mononuclear peripheral blood cells or CCN1(recombinant human pro-angiogenic factor) coating in elderly sheep, emphasizing the importance of cell source and age [30]. Seeking alternatives to conventional prostheses, Driessen-Mol et al. assessed "off-the-shelf" TEHVs using rapidly degrading synthetic scaffolds and vascular-derived cells, observing rapid host cell infiltration and remodeling. However, valve coaptation declined over time, leading to mild-to-moderate regurgitation, highlighting the need for improved stent and valve designs [31]. 

In recent years, ADSCs have attracted attention for their regenerative potential in TEHVs. They enhance tissue integration through paracrine signaling, ECM remodeling, and differentiation into relevant cell types [32]. ADSCs possess strong immunomodulatory and anti-inflammatory properties, releasing growth factors and cytokines such as VEGF, TGF-β, and IL-10, which promote angiogenesis, reduce apoptosis, and mitigate fibrosis, enhancing tissue integration and healing. Moreover, ADSCs inhibit pro-inflammatory immune responses by suppressing the activity of T cells, dendritic cells, and macrophages, while promoting anti-inflammatory cytokine production. This immune regulation reduces inflammation and supports graft acceptance [33].

In our team’s initial attempt, we seeded porcine acellular scaffolds with freshly isolated ADSCs, without prior differentiation or bioreactor conditioning. Harpa et al. found that while acellular xenogeneic valves were stable, non-immunogenic, non-thrombogenic, and non-calcifying, with excellent hemodynamic performance, ADSC-seeding did not enhance regeneration, with signs of right ventricular failure and progressive regurgitation emerging one-month post-implantation. Explanted scaffolds showed host tissue covering the leaflets but lacked cellular infiltration, as most seeded cells died within days, indicating their vulnerability to dynamic pressure and flow [34, 35]. Subsequent *in vitro* studies using bioreactors confirmed significant cell loss under dynamic conditions in seeded scaffolds, emphasizing the need for improved seeding and conditioning techniques to enhance scaffold integration and stem cell survival [36].

### Impact of ADSC-seeding on postoperative complications and survival outcomes

This study represents our team's second attempt at developing living heart valves using ovine allografts. We refined recellularization techniques by differentiating ADSCs into FBs and ECs-like cells, followed by static and dynamic seeding and bioreactor conditioning before orthotopic implantation in pulmonary position in sheep. While previous studies have focused on mechanical properties, biocompatibility, integration, and immunogenicity, our study addresses the gap in understanding age-dependent postoperative complications, survival outcomes, and CPB-related effects in large animal models. We hypothesized that clinical observations, such as fewer postoperative complications, improved recovery and survival might provide indirect evidence of tissue integration and healing. *In vitro* analysis of the mechanical properties of the valves and histological examination of the explants were beyond the scope of this study, as they were addressed by Movileanu et al. [23], part of the same research project. No significant differences in postoperative complications were observed between the DECELL and CELL groups, suggesting that ADSC-seeding did not influence complication rates. However, the CELL group showed a trend toward reduced thoracic drainage (Table 1), and an absence of pleural or pericardial effusions, ascites, and endocarditis (Table 3), likely due to the immunomodulatory and anti-inflammatory properties of ADSCs. On the other hand, findings from Movileanu et al., demonstrated that acellular pulmonary valves in juvenile sheep provided excellent hemodynamics, lacked immunogenic and inflammatory responses, were non-thrombogenic, did not calcify, and provided an effective substrate for cell repopulation. They found that ADSC-seeded valves maintained comparable hemodynamic performance to acellular valves while showing enhanced cellular integration. In the post-implantation phase, valve functionality was serially assessed via echocardiography, confirming normal cusps mobility, coaptation, and valve competence, with no signs of structural failure. Additionally, gross inspection at explantation revealed intact valve leaflets without fibrotic thickening, supporting preserved mechanical integrity. Pre-implantation, TEHVs underwent a preconditioning process in a bioreactor, which gradually increased flow rate, pressure, and frequency to simulate physiological pulmonary circulation. This process allowed us to evaluate several key mechanical properties: (1) Valve opening dynamics: TEHVs demonstrated wide opening with a mean geometrical orifice area of ~ 1.4 cm^2^ for valves with a mean external diameter of ~ 16 mm, confirming proper cusps mobility and flexibility; (2) Closure integrity: The seeded valves achieved perfect central closure with full cusps coaptation, indicating structural stability under pulsatile flow conditions; (3) Tissue resilience: Throughout conditioning, the valves withstood progressive hemodynamic loading without signs of tears, lacerations, or mechanical failure, supporting their durability under physiological stress [23]. Moreover, they noted that ADSC-seeded valves retained leaflet size, suppleness, and functionality for six months post-implantation, with α-smooth muscle actin-positive cells predominantly in the sinus and fibrosa of the leaflets, and endothelial cells covering most surfaces. H&E staining confirmed cellular infiltration, while α-SMA immunohistochemistry showed similar myofibroblast infiltration in ADSC-seeded and DECELL-valves (Base: ~ 40 vs. ~ 35 cells/HPF [cells per high power field]; Mid-leaflet: ~ 30 vs. ~ 25 cells/HPF; Tip: ~ 20 vs. ~ 18 cells/HPF) [23]. The availability of sheep-specific antibodies limited some immunohistochemical assessments, but future research will incorporate broader molecular analyses. Quantitative mechanical testing (e.g., tensile strength, elasticity, and burst pressure) would provide further insights into scaffold durability. In future studies, we plan to perform ex vivo mechanical testing of explanted TEHVs to assess post-implantation structural integrity, to expand bioreactor conditioning studies to include real-time pressure-tracking and force measurements, further validating valve resilience before implantation, and to integrate computational fluid dynamics (CFD) modeling to correlate hemodynamic forces with valve remodeling over time. A larger sample size and extended follow-up, including assessment of inflammatory markers such as IL-6 and TNF-alpha, are needed to better evaluate the role of ADSCs in reducing inflammation, promoting tissue remodeling, and exerting immunomodulatory effects. Additionally, cell tracking techniques would be beneficial to determine whether infiltrating cells originate from the initially seeded ADSCs or are host-derived. These additional analyses will help establish mechanical benchmarks for TEHVs performance and optimize scaffold designs for clinical translation. Systemic inflammation has also been reported as a key factor influencing clinical outcomes in other cardiac pathologies, such as undifferentiated pleomorphic cardiac sarcoma, where elevated inflammatory markers correlated directly with disease activity and prognosis [37].

Our team's results, as previously presented by Harpa et al. and Movileanu et al., highlight the essential role of differentiation, proper seeding, and bioreactor preconditioning in maintaining cell viability and functionality in dynamic environments such as pulmonary circulation [23, 34, 35]. In Movileanu et al. we showed that ADSCs were successfully differentiated into ECs- And FBs-like cells, confirmed by immunofluorescence microscopy for endothelial (CD31, eNOS, von Willebrand factor) and fibroblast (vimentin, Pro-4-hydroxylase, collagen type I) markers. Semi-quantitative analysis showed that ~ 68% of EC-differentiated and ~ 75% of FB-differentiated ADSCs expressed key lineage markers, with a significant marker upregulation in differentiated vs. undifferentiated cells. H&E staining of seeded and preconditioned TEHVs (not implanted) confirmed ECs surface coverage and the presence of FBs at the leaflets base and within the adventitia. These findings confirmed the feasibility of ADSCs differentiation and their regenerative potential [23]. Steinhoff et al. found that static reseeding with autologous myofibroblasts and ECs showed normal valve function up to 3 months in a sheep model of orthotopic pulmonary valve implantation, but histological signs of inflammatory reactions to subvalvar muscle leading to calcifications, were observed also [38]. These findings underscore the importance of dynamic seeding and preconditioning in a bioreactor, as employed in our study, to improve integration and reduce adverse reactions. Also, the use of differentiated FBs and ECs-like in our study aligns with Tudorache et al. [29], where mature endothelial cells demonstrated superior performance compared to incompletely differentiated or undifferentiated cells in studies like Theodoridis et al. [30] and Harpa et al. [34]. The Driessen-Mol study showed that scaffold flexibility and biodegradability influence repopulation and durability [31]. Combined with the anti-inflammatory effects of ADSCs, our results may reflect an interplay between scaffold design and the immunomodulatory properties of the seeded cells, resulting in less postoperative complications and improved survival in CELL group.

Although the six-month survival rate was 57.9%, with no significant differences between the DECELL and CELL groups, the CELL group showed a trend toward higher survival (60% vs. 48.2%) (Fig. 9C). The combined pro-angiogenic, anti-fibrotic and immunomodulatory effects of ADSCs improve vascularization and tissue integration, explaining the observed trend toward better clinical outcomes. The lack of statistical significance in survival rates between valve types (*p* = 0.7768) may be attributed to the fact that both valve types were based on the same decellularized scaffold. The small sample size and relatively short follow-up might not have been sufficient to reveal long-term survival benefits associated with ADSC-seeded valves. Exploring this trend in larger cohorts and a longer follow-up could provide more robust evidence to confirm these findings.

### Age-specific surgical outcomes

Most studies on TEPVs in humans and animals have focused on single age groups, offering limited insights into age-related differences [39, 40]. Our study revealed significantly longer operative times in adult sheep (Fig. 5A, B, C), primarily due to tissue fragility and technical challenges, with aortic wall rupture prolonging procedures by up to 30%. In one case, it resulted in cardiopulmonary arrest, hemorrhagic shock, and intraoperative death, while in two cases, surgical repair was successful without further complications. Age-related changes, such as increased collagen cross-linking, fragmentation and calcification of elastin fibers, contribute to reduced vascular elasticity and increased tissue stiffness [41, 42]. Additionally, aging increases fibrotic tissue formation, altering the biomechanical properties of tissues and making them more prone to mechanical stress and injury, which complicates surgical manipulation in adult sheep, as previously reported [43]. Despite these challenges, our operative times were comparable with those reported by Knirsch et al., who reported CPB times of 65–75 min and total operative times exceeding 200 min [44]. Vis et al. reported longer CPB times (163 min vs. 75 min in our study), likely due to their more complex on-pump on cardiac arrest procedures [40]. Age-based analysis showed no significant differences between groups; however, juvenile sheep experienced more frequent early and late complications (Tables 5 and 6) and reduced survival (Fig. 9B), though not statistically significant. This trend reflects heightened inflammatory responses and poorer outcomes, aligning with pediatric cardiac surgery findings, where younger patients face higher rates of complications such as infections and effusions [45]. Although juveniles may initially appear better suited for these surgeries, the long-term effects of CPB and related stressors contribute to increased late-stage morbidity and mortality. In contrast, adult sheep experienced fewer late-stage complications despite more challenging intraoperative conditions, demonstrating greater resilience to the chronic effects of surgery and CPB, ultimately leading to higher survival rates, a trend also noted in human studies [45].

Although exact data are limited, the perioperative and short-term mortality in sheep undergoing heart surgeries under CPB range from 10 to 33% for the perioperative period (within 48 h) and from 17 to 50% for the first 30 days postoperatively [46]. However, data on long-term survival remain ambiguous, with limited time-specific survival rates. Our study's 30-day survival rate (84.2%) (Fig. 9A) aligns with that reported by Katz et al. (84%) in a similar study [19]. Minimally invasive approaches, such as percutaneous pulmonary valve implantation have shown improved outcomes due to elimination of CPB, reduced inflammatory responses and surgical trauma, leading to faster recovery and improved survival rates. Attmann et al. and Kim et al. reported survival rates of 66.7% over three months in juvenile sheep and a 33.3% mortality rate over six months in adult sheep, respectively, following transcatheter pulmonary valve implantation using self-expanding nitinol valved stents [47, 48]. Our study showed a comparable survival rate of 63.2%. Age-specific surgical strategies and tailored CPB protocols can improve surgical outcomes and survival rates, even without advanced technologies that eliminate the need for CPB. Nevertheless, comparative future studies evaluating conventional CPB-assisted procedures and percutaneous techniques could further optimize surgical protocols and improve long-term outcomes.

### Effects of cardiopulmonary bypass on ovine homeostasis

While the metabolic effects of CPB, such as hyperlactatemia and anemia, are well-documented in humans [3, 49], comparable studies in sheep are scarce. Previous research has focused on specific aspects, such as fetal CPB strategies [50] and baseline physiological changes during sham valve surgeries [51], but age-related CPB effects remain understudied. Our study addresses this gap by providing a comprehensive analysis of age-specific surgical outcomes and CPB-related physiological effects.

CPB leads to physiological changes in blood gas levels and electrolyte imbalances, leading to systemic inflammatory responses and metabolic changes [21]. Human studies have associated elevated lactate levels due to inadequate oxygen delivery and reperfusion injury, with enhanced inflammatory responses, all contributing to unfavorable postoperative outcomes in both adult and pediatric patients [3, 52–55]. Ranucci et al. identified hyperlactatemia during CPB and central venous oxygen saturation (ScVO_2_) < 68% as predictors of increased morbidity and mortality [56]. In our study, prolonged CPB in adult sheep led to significantly higher lactate levels (Fig. 6A, B), metabolic acidosis, and transient anemia, suggesting heightened systemic inflammatory responses Previous studies have also associated prolonged CPB with the accumulation of inflammatory mediators, such as cytokines and acute-phase proteins, exacerbating tissue damage and impairing healing processes [3, 56].

Faustich et al. reported significant decreases in hemoglobin, red blood cell count, hematocrit and albumin post-CPB, attributing these changes to a combination of blood loss, CPB-induced hemodilution, and surgical stress [51], findings consistent with our results. Lower hemoglobin and hematocrit levels in adult sheep during CPB (Fig. 6C, D) highlight their greater susceptibility to CPB-induced anemia, a common complication in cardiac surgery [3]. Hemodilution caused by the priming solution and red blood cell destruction due to non-physiological flow in the ECC circuit are major contributors to anemia in CPB adult models [2, 57]. In contrast, juvenile sheep exhibited a stronger hematopoietic response and better adaptation to CPB stress. Over the 6-month follow-up, three deaths in juvenile sheep with decellularized valves were attributed to endocarditis, likely due to the heightened inflammatory response following CPB and increased susceptibility to bacterial adhesion, as previously reported [22]. Although ADSC-seeding did not significantly reduce acute surgical risks, our observations emphasize the need for strategies to mitigate late-stage inflammatory complications, aligning with findings from other small animal and human studies [2, 3].

### Coagulation homeostasis

This study provides insights into the age-specific anticoagulation management, focusing on heparin dosing, pre- And post-CPB ACT values, and protamine doses. Thrombosis of the ECC circuit can be fatal for patients, while inadequate control may contribute to postoperative hemorrhage, a major cause of morbidity and mortality in cardiac surgery [58]. ACT was monitored intraoperatively every 30 min to assess the adequacy of the heparin regimen, with reference values between 80 and 120 s [59].

Juvenile sheep required significantly higher intravenous heparin doses (Fig. 8A, B) to achieve adequate anticoagulation, likely due to faster metabolism and clearance rates, while ECC-administered doses remained similar across age groups, bypassing metabolic variability. The significant difference in pre-CPB ACT (Fig. 8C) suggest age-specific baseline coagulation profiles, with juveniles requiring more time to reach target ACT levels, as previously reported [2, 3]. The heparin-to-protamine ratio was effectively managed in both groups, maintaining adequate coagulation without excessive bleeding or thrombotic events. Despite initial pre-CPB ACT differences, post-CPB ACT values were comparable (Fig. 8D), indicating similar coagulation normalization and effective heparin reversal. The greater variability in post-CPB ACT in juveniles, reflected by a higher standard deviation, may be attributed to age-related physiological differences or individualized responses to protamine, as described in previous studies [2, 60, 61]. 

Unlike previous studies that focused on single age cohorts [29–31, 34, 35, 38, 40], our study demonstrates clear age-specific differences in surgical outcomes and anticoagulation management, emphasizing the distinct effects of CPB on juvenile and adult sheep. To the best of our knowledge, this is the first study to comprehensively analyze these physiological and hematological differences during heart surgeries. The observed variability between age groups underscores the need for tailored CPB and coagulation protocols to optimize outcomes and address age-related challenges.

### Key findings and clinical implications

We have summarized the key findings and the broader implications of this study in Table 10. Ultimately, achieving a truly living heart valve that fulfils all performance criteria—including hemodynamic functionality, durability, and the ability to grow and self-repair—while also being readily available off-the-shelf, requires further research. This includes identifying optimal stem cell sources, refining recellularization techniques, and developing efficient seeding methods to enhance scaffold integration and regeneration. The development of more standardized seeding and conditioning protocols is necessary to improve batch-to-batch reproducibility, a crucial factor for eventual clinical translation and regulatory approval. Continued advancements in this field are essential to bridge the gap between the two approaches—acellular vs. seeded scaffolds—in order to achieve the next generation of TEHVs. Comparative studies between cell-seeded and cell-free scaffolds will help clarify whether endogenous repopulation alone is sufficient for long-term valve function, a question of significant clinical importance given that cell-free approaches have gained traction in regenerative medicine. Cell-tracking strategies would be beneficial to determine whether implanted ADSCs contribute to tissue remodeling or if host-derived cells primarily drive neotissue formation. Larger preclinical studies and long-term functional validation—extending follow-up beyond six months to assess chronic inflammation, neotissue formation, and valve function—along with careful consideration of manufacturing and regulatory pathways, will be essential for progressing toward clinical trials and market translation. By addressing these translational challenges, TEPVs could eventually provide an off-the-shelf solution for congenital and acquired pulmonary valve disease, improving outcomes for pediatric and adult patients alike.

**Table 10:** Key findings and the broader implications of this study

Aspect	Key findings	Implications
Age-specific surgical Strategies	-Adult sheep—greater intraoperative challenges but better long-term survival -Juvenile sheep—easier surgeries but poorer long-term survival	-Adult sheep—better suited for chronic experimental studies using conventional CPB-assisted procedures -Juveniles—better suited for acute, short-term experiments -Future comparative studies—conventional CPB-assisted procedures vs. percutaneous techniques to optimize surgical protocols and improve long-term outcomes
Age-SpecificCPB-related physiological effects	-Adult sheep—greater metabolic stress during CPB -Juveniles—more resilient to early CPB stress but more prone to late-stage complications post-CPB	-Tailoring age-specific surgical strategies and CPB protocols—improved surgical outcomes and survival rates -Juveniles—need strategies to mitigate late-stage complications
ADSCs impact on postoperative complications and Survival	-ADSC-seeding—no significant impact on survival rates or complications (by indirect evidence through clinical observation) -CELL group—improved surgical outcomes and survival rate (higher trends) -Comparison between DECELL and CELL—complicated by variability from different age groups	-Benefits of ADSC-seeding in reducing complications and enhancing survival—further studies with larger cohorts and extended follow-up -Clearer insights into ADSC-seeding effects—achievable by reducing variability with a more homogeneous age group
Age-specific coagulation protocol	-Juvenile sheep—higher heparin doses for adequate coagulation due to faster metabolism and clearance rates -Juveniles—increased variability in ACT normalization due to age-specific baseline coagulation profiles or individualized protamine responses	-Individualized anticoagulation protocols—reducing variability and improving outcomes in juvenile sheep

### Study limitations

Several limitations of this study were identified: (1) The small sample size may have been insufficient to detect subtle differences in postoperative complications or mortality, particularly regarding the effects of ADSC-seeding. To adhere to ethical principles and the 3R framework (replacement, reduction, and refinement), the number of animals was deliberately minimized; (2) Age distribution: The inclusion of both juvenile and adult sheep introduced variability, potentially complicating comparisons between the DECELL and CELL groups. A more homogeneous age group could have reduced this variability, providing clearer insights into the specific effects of ADSC-seeding; (3) Follow-up period: The study focused on short- and medium-term surgical outcomes and physiological changes associated with CPB, with a follow-up period limited to six months. A longer observation period would provide valuable insights into chronic complications, and the long-term effects of ADSC-seeded valves on tissue integration and regeneration; (4) Detailed *in vitro* analysis including mechanical testing (tensile strength, elasticity, burst pressure) and expanded bioreactor-based assessments, are planned for future studies, to further validate TEPVs structural integrity and long-term performance. *In vitro* differentiation assays of TEHVs pre-implantation and histological examination of the explanted valves were beyond the scope of this study; therefore, the mechanisms of ADSC-mediated tissue integration were not directly assessed. These aspects will be addressed in upcoming research; (5) The absence of quantified inflammatory markers, such as IL-6 and TNF-alpha would provide a more precise assessment of the inflammatory response. Future studies will incorporate comprehensive inflammatory profiling to better elucidate the role of ADSCs in modulating immune responses and inflammation.

In conclusion, the following can be affirmed:

Our study demonstrates that age significantly impacts surgical complexity and outcomes. Adult sheep, despite greater intraoperative challenges, demonstrated better long-term survival, making them more suitable for chronic experimental studies. Juvenile sheep, while undergoing less demanding procedures, experienced more late-stage complications, indicating their suitability for short-term studies in conventional CPB-assisted procedures.

Cardiopulmonary bypass significantly affected metabolic markers, with adult sheep showing a greater tendency for metabolic acidosis and anemia, reflecting increased metabolic stress. The higher mortality rate in juvenile, suggests an increased inflammatory response post-CPB, emphasizing the need for age-specific surgical strategies and CPB protocols.

ADSC-seeding did not significantly impact operative parameters, complications or survival rates. Further research with an extended follow-up period is needed to better understand the long-term effects of ADSC-seeding on tissue integration and regeneration.

Our findings highlight the importance of age-specific anticoagulation protocols for optimizing perioperative management and preventing complications. While CPB-related coagulopathy was effectively managed across age groups, individualized anticoagulation strategies for juveniles could further minimize variability in postoperative coagulation responses.

Therefore, valve type alone cannot be sufficient to address the complexities of postoperative recovery, particularly in the context of age-related variability and CPB-induced metabolic stress.

## Data Availability

The datasets used and/or analyzed during the current study are available from the corresponding author on reasonable request.
